# Efficacy and Safety of Ultrasound-Guided Percutaneous Polidocanol Sclerotherapy in Benign Cystic Thyroid Nodules: Preliminary Results

**DOI:** 10.1155/2017/8043429

**Published:** 2017-05-31

**Authors:** Xiaohua Gong, Qi Zhou, Fang Wang, Wenjun Wu, Xiaojun Chen

**Affiliations:** ^1^Department of Endocrinology and Metabolism, The First Affiliated Hospital of Wenzhou Medical University, Ouhai District, Wenzhou, Zhejiang 325015, China; ^2^Department of Pathology, The First Affiliated Hospital of Wenzhou Medical University, Ouhai District, Wenzhou, Zhejiang 325015, China

## Abstract

**Objective:**

To evaluate the efficacy and safety of percutaneous polidocanol injection (PPI) in treating cystic thyroid nodules.

**Materials and Methods:**

A total of 158 cystic or predominantly cystic thyroid nodules (>80% cystic component) in 143 patients were evaluated. 114 patients with compressive symptoms or aesthetic complaints were offered PPI. 44 individuals without compressive symptoms and aesthetic complaints who were only followed up clinically were used as the control group. The efficacy and safety of PPI were evaluated for 1 month, 3 months, 6 months, 9 months, and 12 months of follow-up.

**Results:**

In the PPI group, the mean baseline volume of 15.6 ± 18.9 cm^3^ reduced at the 1-month follow-up to 5.1 ± 5.6 cm^3^ (*p* < 0.001) and 0.6 ± 0.9 (*p* < 0.001), and nodules shrunk according to the time after PPI (*p* < 0.001). A complete response (if ≥70% decrease) to PPI at the 12-month follow-up occurred in 100% of the cystic or predominant cystic nodules. None of the nodules recurred at the 12-month follow-up after PPI. The side effects were mild. Twenty patients (17.5%) developed mild localized pain, and fourteen cases (12.3%) experienced mild or moderate fever after PPI.

**Conclusions:**

PPI is a safe and effective alternative to treat benign cystic or predominant cystic thyroid nodules.

## 1. Introduction

Thyroid nodules are characterized by excessive structural growth, functional transformation, and/or cystic degeneration of one or several areas within the gland [1]. According to various studies, 15–30% of thyroid nodules are cystic or predominantly cystic [2, 3]. After simple fine-needle aspiration, most cystic lesions (around 80%) refill and enlarge over time [4]. Surgery is a long-established therapeutic option for benign thyroid nodules. However, the cost of thyroid surgery, risk of temporary or permanent complications, and impact on quality of life remain relevant concerns. Ethanol ablation (EA) is an effective and safe treatment technique [5]. However, in a randomized study, percutaneous ethanol injection (PEI) achieved remission in 68% of cystic thyroid nodules after one treatment injection [4]. Ethanol sclerotherapy is also associated with some complications. Mild transient pain or a burning sensation at the site of injection is the most commonly seen following ethanol sclerotherapy and is a result of leakage of ethanol into the subcutaneous tissue [4, 6]. Other uncommon complications include hematoma, dyspnea, and vocal cord paralysis [7, 8]. More importantly, it is difficult to obtain the 99% ethanol in China. Therefore, there is a need for novel sclerosants. Polidocanol injection is well known as a novel foam sclerosant that is currently administered by injection mostly for the treatment of hemangiomas [9], haemorrhoidal disease [10], symptomatic hepatic cysts [11], gastric varices [12], and reticular veins of the lower limbs [13]. The aim of the present study was to prospectively determine the efficacy and safety of polidocanol injection for the treatment of benign cystic or predominant cystic thyroid nodules.

## 2. Materials and Methods

From October 2013 to April 2015, a total of 117 cystic (>90% cystic component) and 41 predominantly cystic thyroid nodules (>80% cystic component) in 143 patients (128 patients had single nodule, and 15 patients had two cystic or predominantly cystic nodules) were evaluated with fine-needle aspiration cytology (FNAC), using 21 G hypodermic needles at the outpatient clinic of The First Affiliated Hospital of Wenzhou Medical University. And complete fluid aspiration was performed at this time. Benign cytological results were from two separate US-guided fine-needle aspirations to exclude malignancy. 114 consecutive patients with compressive symptoms or aesthetic complaints were offered polidocanol treatment. 44 individuals without compressive symptoms and aesthetic complaints who were only followed clinically were used as the control group. This prospective study was conducted over a period of one year.

US examination was performed in all patients before treatment and at the time of the 1, 3, 6, 9, and 12 months of follow-up examinations. The three orthogonal diameters of each nodule that were measured were the largest diameter and two mutually perpendicular diameters. The volume of each nodule was calculated using the equation volume = *π* *abc*/6, where *a* is the largest diameter and *b* and *c* are the other mutually perpendicular diameters. Upon US examination, the changes in the volume of the nodules were evaluated.

Before and after PPI treatment, the patients were asked to rate pressure symptom score on a visual analog scale (0–10 cm) and cosmetic grade ((1) no palpable mass, (2) a palpable mass but no cosmetic problem, (3) cosmetic problem on swallowing only, and (4) readily detected cosmetic problem) [14].

To assess safety, any possible complications both during and immediately after the procedure were evaluated. Major and minor complications were defined by the definitions of the Society of Interventional Radiology [15]. Procedure-related pain was graded into four categories (grade 0, no pain or mild pain similar to pain during lidocaine injection; grade 1, pain greater than lidocaine injection, but not needing medication; grade 2, pain needing medication; grade 3, PPI procedure incompletely terminated due to severe pain) [14].

According to the decrease in nodular volume, polidocanol results were categorized as incomplete response (if <70% decrease) or complete response (if ≥70% decrease) [16].

**Figure 1:**
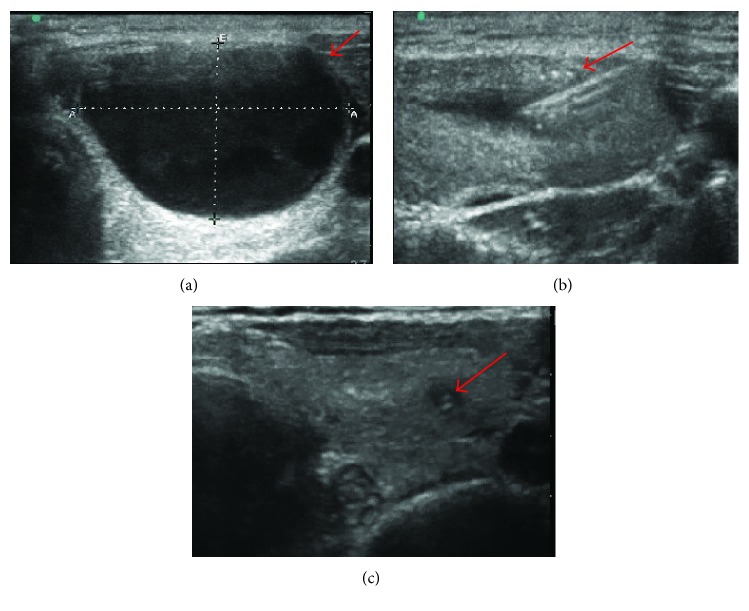
Ultrasonographic images of a 23-year-old woman before, during, and after treatment: (a) a cystic thyroid nodule of ultrasonographic images before PPI treatment; (b) during PPI treatment; (c) 12 months after PPI treatment; the nodule decreased significantly (arrow). PPI: percutaneous polidocanol injection.

The procedures were performed in the outpatient clinic. Patients were placed in a supine position with mild neck extension. An 18-gauge needle was inserted into the cystic portion of the thyroid nodule under ultrasound guidance without local anesthesia. The fluid was first completely aspirated. The syringe tube was then replaced with another one containing polidocanol injection while keeping the puncture needle in place. The amount of polidocanol injected was about 25–33% of the amount of fluid aspirated. Then, we extracted and flushed the injection for 5 times to assure the uniform distribution of the injection and the polidocanol was left in the cysts (Figure 1).

This study was approved by the Ethics Committee of The First Affiliated Hospital of Wenzhou Medical University. All study participants provided written informed consent before the experiments. The written informed consent was provided by each eligible patient, and the study conformed to the Declaration of Helsinki.

## 3. Statistical Analysis

The comparison of parametric quantitative variables between both groups was analyzed with Student's *t*-test and nonparametric variables with the Mann–Whitney test. Qualitative variables were evaluated with the chi-square test. The comparison of volumes at different periods was conducted with the Friedman test, confirmed with nonparametric multiple comparison for dependent data. The same comparison in the control group was conducted with the paired Wilcoxon test. We applied the likelihood ratio to compare complete or incomplete response rates after polidocanol treatment using the chi-square test. Multivariate analysis was performed with the Statistical Package for Social Sciences (SPSS), version 19.0. We used a confidence interval of 95% and a significance level of 5%.

## 4. Results

The clinical data for 114 consecutive patients with polidocanol treatment and 44 individuals in the control group are given in Table 1. At baseline, the volume of the nodules in the polidocanol treatment group ranged from 2.5 to 138.7 cm^3^, with an average of 15.6 ± 18.9 cm^3^. In the control group, the initial volume of the nodules ranged from 0.9 to 14.2 cm^3^ (mean 2.3 ± 3.0 cm^3^). After a follow-up of 12 months, the volumes in the control group ranged from 0.4 to 16.4 cm^3^ (mean 2.8 ± 3.5 cm^3^, *p* = 0.501).

**Figure 2:**
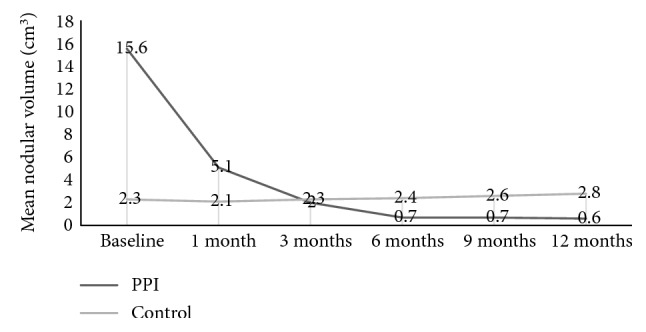
Volumes at baseline and the time of follow-up in the PPI group; volumes at baseline and the time of follow-up in the control group. PPI: percutaneous polidocanol injection.

At 1-month follow-up, the volume of the nodules in PPI group ranged from 0.2 to 31.30 cm^3^, with an average of 5.1 ± 5.6 cm^3^. At 3 months, 6 months, and 9 months of follow-up, the cyst nodules showed significant reduction in the volume. The mean volumes of nodules at the 3 months, 6 months, and 9 months of follow-up were 2.0 ± 3.8 cm^3^, 0.7 ± 1.0 cm^3^, and 0.7 ± 0.9 cm^3^, respectively. At 12-month follow-up, the nodules ranged in volume from 0.01 to 4.9 cm^3^, with an average of 0.6 ± 0.9 cm^3^. Compared with the baseline volume, the reduction after PPI treatment showed a significant difference (*p* < 0.01, Figure 2).

Compared with the volume at 1-month follow-up, the reduction at 12-month follow-up was also significantly different (*p* < 0.01, Figure 2). Compared with the outcomes between both groups, we observed that the nodules in the PPI group showed a significantly larger reduction in volume (*p* < 0.001, Figure 2).

Compared with baseline, a reduction at 1-month follow-up was 65.11 ± 23.01% (*p* < 0.001, Table 2). Compared with the volume at 1-month follow-up, the reduction at 12-month follow-up showed a significant difference (*p* < 0.001, Table 2). The therapeutic success rate to PPI at 1-month follow-up occurred in 36.8% of the cystic or predominant cystic nodules. At 3-month follow-up, the therapeutic success rate to PPI was observed in 89.5% of the nodules, 98.2% of the nodules at 6-month follow-up, and 100% of the nodules at 12-month follow-up (*p* for comparison between all the follow‒ups < 0.001). Mean symptom score and cosmetic grade also significantly vanished in the vast majority at last follow-up of PPI group (*p* < 0.001, Table 2).

A comparison of the results between baseline and post-PPI showed no statistical difference in TSH (*p* = 0.579) or free T4 (*p* = 0.621) levels.

Twenty of the 114 (17.5%) patients after PPI treatment reported mild localized pain that lingered for 1 to 3 days. Fourteen cases of the 114 (12.3%) patients experienced mild or moderate fever after polidocanol injection. The fever lingered for 1–4 days that required no treatment or antipyretic drug. None suffered the major complications in this study.

## 5. Discussion

Simple aspiration is the treatment of choice for diagnostic and therapeutic purposes in symptomatic patients with cystic or predominant cystic thyroid nodules. However, the recurrence rate is up to 80% [17], even after repeated aspirations. Success rate of PEI, defined as near disappearance or marked size reduction (>50%), has been reported to vary from 72% to 95% [4, 8, 16]. PEI remains the first line treatment for pure thyroid cysts or predominantly cystic (cystic components comprising > 80%) benign nodules [18, 19]. However, a prospective randomized double-blind study showed that the cure was obtained in 64% of patients after one treatment of PEI sclerotherapy only and the other patients needed one more treatment session [4]. Furthermore, side effects related to ethanol seepage outside the capsule may cause pronounced pain or more serious side effects such as paresis of the vocal cords or extraglandular fibrosis, which may impede subsequent surgery in case of treatment failure [7]. Radiofrequency (RAF) is scarcely employed in the management of cystic thyroid lesions. Although both ethanol ablation and RFA are effective and safe treatment techniques, ethanol ablation is a less expensive and simpler procedure than RFA [20].

It is difficult to obtain the 99% ethanol used as a sclerosant in China because the absolute ethanol production has been disrupted for four years, and hospital-made absolute ethanol cannot obtain the registered document of approval. Polidocanol (Polidocanol®) is an effective sclerosant consisting of 95% hydroxypolyethoxydodecane and 5% ethyl alcohol. The Food and Drug Administration (FDA) has approved polidocanol as a medicinal sclerosant in the United States, as it has its equivalents in European countries.

Although there are many studies in other areas, few studies have examined polidocanol as a sclerosant for cystic or predominant cystic thyroid nodules. In our present study, the rate of volume reduction of the nodules was 95.58% 12 months after PPI. Moreover, we achieved a 100% therapeutic success (size reduction > 70%) in 114 patients and none of the nodules recurred at 12-month follow-up. The observed success rate was better than those reported previously PEI [14, 21]. And symptom scores and cosmetic scores vanished in the vast majority. The mechanism of treating cyst is probably destroying endothelial cells of capsule wall which causes aseptic inflammation; thus endothelial tissue atrophies and cyst cavity adhere and occlude [22]. A rabbit model histopathologic evaluation results revealed that inflammation and fibrosis were significantly increased. This finding shows that polidocanol can be effectively used to treat thyroid nodules by means of fibrosis [23]. Meanwhile, it has anesthetic effects, no need for intracystic anesthesia or sedation for the patients [24]. The primary clinical application of polidocanol is its endovascular use for sclerosing varicose veins, and, therefore, it is a safer agent for using without reaspiration. It can be left in the cyst cavity to achieve a complete adhesion with only a one-step application. So its procedure was easier than PEI.

In our experience, mild pain was only seen in 17.5% of patients and mild or moderate fever was seen in 12.3% patients treated with PPI. There were no serious side effects related to ethanol seepage outside the capsule.

The present study had some limitations. We did not compare PPI treatment with a control group treated with ethanol because we could not obtain absolute ethanol. Secondly, the patients were followed up only for one year. Further studies with larger patient samples are needed to confirm these findings.

The findings of this study indicate that PPI is a safe, effective, and rapid treatment that can be performed on an outpatient basis. The cost of PPI treatment for benign cystic or predominant cystic thyroid nodule is 600 RMB, and the cost of PEI treatment was 260 RMB. Although PPI has a higher cost than PEI, it is still inexpensive. In short, it may be an effective alternative to ethanol injection.

## Figures and Tables

**Table 1:** Clinical characteristics of patients in the PPI and control groups.

	PPI (*n* = 114)	Control (*n* = 44)	*p* value
Gender	72F/42M	26F/18M	0.29
Age (years)	48.63 ± 12.73	45.21 ± 11.18	0.20
Baseline volume (cm^3^)	15.6 ± 18.9	2.3 ± 3.0	<0.001
Ultrasound findings			0.83
Cystic (*n*)	59	25	
Predominantly cystic (*n*)	55	19	

PPI: percutaneous polidocanol injection; F: female; M: male.

**Table 2:** Efficacy of PPI in cystic thyroid nodules at follow-up.

	Baseline	1 month	3 months	6 months	12 months	*p*
Size reduction (%)	—	65.11 ± 23.01	85.87 ± 14.45	94.75 ± 5.76	95.58 ± 4.18	<0.001
Success rate, *n* (%)	—	42/114 (36.8)	102/114 (89.5)	112/114 (98.2)	114/114 (100)	<0.001
Symptom score	4.71 ± 1.62	2.34 ± 0.96	1.12 ± 0.83	0.21 ± 0.40	0.21 ± 0.40	<0.001
Cosmetic grade	3.49 ± 0.50	2.05 ± 0.64	1.65 ± 0.53	1.37 ± 0.50	1.21 ± 0.41	<0.001

PPI: percutaneous polidocanol injection.
